# Medication literacy among community-dwelling older adults in Shenzhen: a 2024 cross-sectional survey with psychometric evidence

**DOI:** 10.3389/fpubh.2026.1728367

**Published:** 2026-03-16

**Authors:** Fenfang Wei, Menghuan Yang, Xiaoyu Liu, Qian Wang, Jianru Wu, Qianhe Jian, Wenyu Wu, Mengdan Xu

**Affiliations:** 1 Shenzhen Institute of Pharmacovigilance and Risk Management, Shenzhen, China; 2 School of Pharmacy, Guangdong Pharmaceutical University, Guangzhou, China; 3 National Medical Products Administration Key Laboratory for Technology Research and Evaluation of Pharmacovigilance, Guangzhou, China

**Keywords:** medication literacy, influencing factors, older adults, functional literacy, communication literacy, critical literacy, Shenzhen

## Abstract

**Objective:**

To provide a scientific basis for policymakers and healthcare providers in developing targeted interventions, we investigated the level of medication literacy and its contributing factors among the older population in Shenzhen.

**Methods:**

A cross-sectional study was conducted between August and October 2024, involving 1,198 participants aged 60 years or older from 10 administrative districts in Shenzhen, using a multi-stage stratified random sampling method. The survey instrument comprised 20 items, grouped into three dimensions: functional literacy, communication literacy, and critical literacy. Data were analyzed using descriptive statistics, chi-square tests, and multiple linear regression with prespecified covariates to explore the status of medication literacy and its associated factors among older adults in Shenzhen communities.

**Results:**

A total of 1,005 valid questionnaires were collected. The average scores for functional literacy, communication literacy, and critical literacy of older adults in Shenzhen were 1.44 ± 0.37, 1.20 ± 0.35, and 0.85 ± 0.26, respectively. The overall average score was 69.71 ± 16.57. Low medication literacy was the most prevalent (*n* = 407, 40.50%), followed by medium (*n* = 366, 36.42%) and high literacy (*n* = 232, 23.08%). Significant factors associated with medication literacy among older adults included educational level, type of medical insurance, disease duration, frequency of hospitalizations in the past year, ADR history, and the situation of contacting medical staff. All of these factors were statistically significant (*p* < 0.05).

**Conclusion:**

The medication literacy of older adults in Shenzhen requires more attention, targeted education is warranted for subgroups with low education, no medical insurance, short disease duration, three or more hospitalizations in the past year, low awareness of ADRs, and less contact with medical staff.

## 1 Introduction

As is well known, drugs are important means for treating diseases and improving health. With the high incidence of chronic diseases (1) and the prevalence of polypharmacy (2), rational use of drugs has become particularly important. However, insufficient medication literacy remains a global problem that not only affects drug efficacy but also poses a threat to medication safety (3, 4). Medication literacy refers to the ability of individuals to obtain, understand, communicate, calculate, and process relevant specific drug information, make rational drug and health decisions, and use drugs safely and effectively (5). Medication literacy reflects not only patients’ medication compliance but also their ability to use drugs safely, and it thus has a significant impact on ensuring their safe and effective use of drugs (6). Elizabeth et al. showed that obtaining adequate health literacy can help individuals to correctly understand information about their health conditions and the drugs needed for treatment, which is essential to maintain medication adherence (7). Investigations into medication literacy and self-management among inpatients taking multiple drugs have revealed that medication literacy serves as an important predictor of medication self-management. Higher levels of medication literacy are associated with improved self-management skills and better medication adherence in patients (8). In addition, studies have shown that improving medication literacy can significantly improve treatment outcomes and reduce readmission rates and medical costs (9). Therefore, enhancing public medication literacy is a critical factor in improving overall health outcomes and healthcare quality. China is becoming a rapidly aging society (10), and the older adults are the population with the highest proportion of chronic diseases and greatest challenges with drug use (11). Yet, a research gap currently remains regarding the assessment data and influencing factors of medication literacy among the older adults across Shenzhen, This study aims to estimate medication-literacy levels and identify independent sociodemographic and care-access predictors among Shenzhen elders.

## 2 Methods

### 2.1 Participants

This study was approved by the Ethics Review Committee of the First Affiliated Hospital/The First Clinical Medicine School of Guangdong Pharmaceutical University (the ethics approval number was 2024IIT98). Before distributing the questionnaires, a detailed introduction was provided to the participants about the purpose and content of the study. Based on the principles of voluntary and anonymous participation, the participants were informed at the beginning of the questionnaire that they had the right to withdraw at any time during the questionnaire filling process, and that this would not harm their related interests. A multi-stage, stratified, random sampling method was used to conduct a cross-sectional survey from August to October, 2024. All 10 administrative districts of Shenzhen were included in the sampling range. Based on consideration of the seventh population census data of Shenzhen and the target sample size, the sample size for each administrative district was determined based on the proportion of the older population in that district to the total older population of the entire city of Shenzhen. Three to four sample communities were then randomly selected using systematic sampling method from each administrative district, and within each selected community, a specified number of older adult individuals are drawn from the roster of all older adults in that community to participate in the survey using simple random sampling by members of the research team and social workers who had been uniformly trained in each sample community. The questionnaires were completed by face-to-face interviews with the participants. The inclusion criteria for the participants were defined as follows: (1) permanently residing in Shenzhen for more than 6 months; (2) age ≥60 years; (3) voluntary participation in the questionnaire survey. The exclusion criterion was a hearing, vision, cognitive, or other impairment that prevented cooperation in the investigation.

### 2.2 Sample size calculation

The sample size was calculated according to the following cross-sectional survey sample size calculation formula:


$$N=\frac{{Z_{a/2}^{2}}^{*}p^{*}(1-p)}{d^{2}}$$

**Figure 1:**
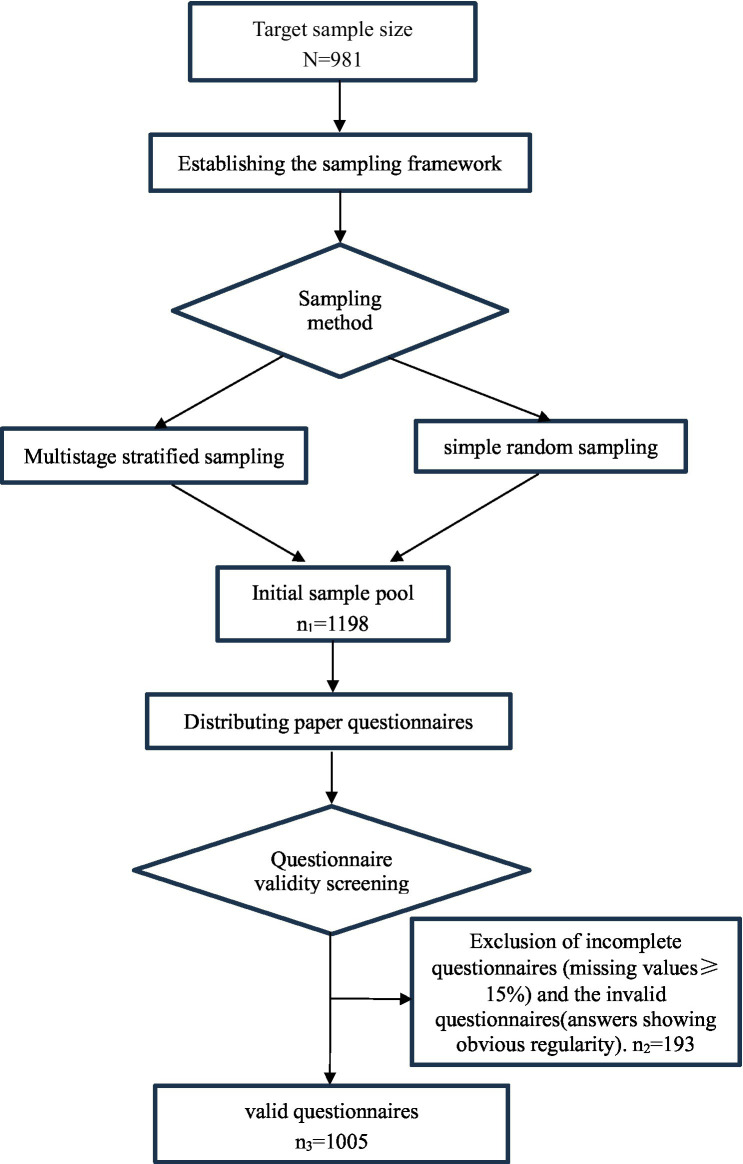
The participant-flow figure.

Taking a two-sided *α* value of 0.05 and $Z_{a/2}=1.96$ , referring to the 29.70% health literacy rate of Chinese residents in 2023, the *p*-value in this study was 0.297, and the D-tolerance error was set at 0.03. The minimum sample size required for this study was 892, and considering an invalid rate of 10%, the target sample size was at least 981. Therefore, a total of 1,198 questionnaires were distributed, after excluding the incomplete questionnaires with missing values≥15% and invalid questionnaires (answers showing obvious regularity), 1,005 valid questionnaires were finally included in this study, with an effective recovery rate of 83.89%. The participant-flow figure was as follow (Figure 1).

### 2.3 Survey instrument

#### 2.3.1 Questionnaire design

The survey consisted of two parts: an impact factor questionnaire and a medication literacy scale. The impact factor questionnaire gathered information on participants’ demographic characteristics, including district, gender, age, education level, marital status, living status, and disease-related information. ased on the concept of medication literacy proposed by Neiva (12) and referring to mature assessment tools at home and abroad, the initial index system item pool of medication literacy scale was constructed. After two rounds of Delphi expert consultation and pre-survey items screening, the revised medication literacy scale for older adults was developed with three dimensions: functional literacy, communication literacy, and critical literacy. The scale consisted of 20 items in total: 13 items measured functional literacy, 4 items assessed communication literacy, and 3 items evaluated critical literacy. The weights of each primary and secondary indicators were obtained also through the Delphi expert consultation method (Table 1).

**Table 1:** Medication literacy assessment index system (including weights).

Primary indicators (weights)	Secondary indicators (weights)
Functional literacy (0.392)	GN1 I can understand the contents such as the medication instructions and labels. (0.0343)
	GN2 I understand that storing medicines in accordance with the requirements of the drug instructions can prevent them from deteriorating and losing efficacy. (0.0317)
	GN3 I can follow the instructions on the label or the drug instructions to take the correct dosage (such as the number of pills, drops, volume, etc.) (0.0343)
	GN4 I can distinguish between prescription drugs and over-the-counter drugs that I use. (0.0211)
	GN5 I know the correct method of using each medication I take (such as the proper time to take the medication and the interval between doses). (0.0338)
	GN6 I am clear about the therapeutic purpose of each medication I use. (0.0298)
	GN7 I can name every medication I use. (0.0258)
	GN8 I am clear about the main side effects of my commonly used medications (such as headache, nausea, diarrhea, dizziness, etc.) (0.0280)
	GN9 I know what dietary restrictions are needed during medication use (0.0269)
	GN10 I can clearly read the text on the drug instructions and the medication administration labels. (0.0349)
	GN11 I can obtain drug information through various channels (such as drug instructions, medication administration labels, television, promotional brochures, lectures, the internet, etc.) (0.0282)
	GN12 I am able to correctly understand drug information with the help of external parties such as medical personnel, relatives, and friends. (0.0294)
	GN13 I can clearly inform the doctor about my medication situation (such as drug names, dosage, time, etc.) (0.0338)
Communication literacy (0.350)	JL1 When I feel that my symptoms have changed, I will promptly consult a doctor to determine if medication adjustment is necessary. (0.0929)
	JL2 If I do not understand the medical staff’s explanation of the medication, I will keep asking questions until I get a clear understanding. (0.0866)
	JL3 If I have stopped taking medication or changed the dosage, or am considering doing so, I will inform my doctor or family members in a timely manner. (0.0858)
	JL4 When I want to know more information about the drug I’m concerned about, I will consult medical staff. (0.0848)
Critical literacy (0.258)	PP1 I would question the authenticity and reliability of drug information seen on television, advertisements, social media (such as WeChat, Douyin, etc.). (0.0819)
	PP2 I will monitor my own health status (such as blood pressure, blood glucose levels, etc.) to assess the effectiveness of medication. (0.0902)
	PP3 Before taking medication, I will check the drug’s expiration date and appearance quality to determine if it is suitable for use. (0.0858)

Statistical analysis showed that the overall Cronbach’s α coefficient of the revised medication literacy scale was 0.951, and the split-half coefficient was 0.964, both of which were higher than 0.900, indicating that the index system was very reliable. Results of the exploratory factor analysis (EFA) showed that KMO value was 0.956, Bartlett *χ*^2^ = 7397.127 (*p* = 0.0000). Confirmatory factor analysis (CFA) showed that CMIN/DF = 3.721 (<5.000), RMSEA = 0.074 (<0.080), IFI = 0.940 (>0.900), CFI = 0.940 (>0.900), NFI = 0.920 (>0.900), TLI = 0.930 (>0.900), indicating that the questionnaire model had good fit validity. The factor loadings of each item for the three dimensions ranged from 0.666 to 0.874, and the AVE values of each dimension ranged from 0.560 to 0.655, both of which were greater than 0.500; the composite reliability (CR) values ranged from 0.790 to 0.952, all greater than 0.700, indicating that the scale had good convergent validity.

#### 2.3.2 Questionnaire scoring

The questionnaire items were scored using a 5-point Likert scale. The items for the functional literacy dimension replaced “not at all” “rarely” “generally” “mostly” and “completely” with a numerical value ranging from 1 to 5, respectively, and the degree increased in turn. The items for the dimensions of communication literacy and critical literacy were replaced by the numbers 1 through 5, representing “never” “occasionally” “sometimes” “often” and “always” respectively, with the frequency increasing sequentially. The total score of the questionnaire ranged from 20 to 100, with higher scores representing higher levels of medication literacy.

The calculation formula for the comprehensive medication literacy score was as follows:


$$S=20\cdot \sum_{i=1}^{20}\bar{\mathrm{Ai}}\cdot \mathrm{Xi}$$

*S* represented the comprehensive evaluation score of the respondents’ medication literacy, $\bar{\mathrm{Ai}}$ represented the average score given by the 1,005 respondents for the *i*-th secondary indicator, and *Xi* represented the weight value of the *i*-th secondary indicator (Table 1). This result *S* was a score converted to a hundred-point scale.

The calculation formula for the mean scores of the functional literacy dimension, communication literacy dimension, critical literacy dimension were as follows:


$$S1=\frac{\left(\sum_{j=1}^{1005}\sum_{i=1}^{13}\mathrm{GN}i^{*}\mathrm{Xi}\right)}{1005}$$

S1 represented the average score of the functional literacy dimension of the survey subjects, *GNi* represented the score given by the survey subjects for the *i*-th secondary indicator, *Xi* represented the weight value of the *i*-th secondary indicator.


$$S2=\frac{\left(\sum_{j=1}^{1005}\sum_{i=1}^{4}\mathrm{JL}i^{*}\mathrm{Xi}\right)}{1005}$$

*S2* represented the average score of the communication literacy dimension of the survey subjects, *JLi* represented the score given by the survey subjects for the *i*-th secondary indicator, *Xi* represented the weight value of the *i*-th secondary indicator.


$$S3=\frac{\left(\sum_{j=1}^{1005}\sum_{i=1}^{3}\mathrm{PP}i^{*}\mathrm{Xi}\right)}{1005}$$

*S3* represented the average score of the critical literacy dimension of the survey subjects, *PPi* represented the score given by the survey subjects for the *i*-th secondary indicator, *Xi* represented the weight value of the *i*-th secondary indicator.

### 2.4 Quality control

Prior to the survey administration, the research team members participated in a unified training session to familiarize themselves with the content of the questionnaire, and participants were informed about the purpose of the study and were assured of its anonymous and voluntary nature. The investigators then ensured that the questionnaires were completed by the participants independently. For any participants who experienced difficulties reading any items on the questionnaire, the investigators explained the items with a neutral attitude to ensure that the participants could understand the questions and answer them independently. All questionnaires were collected by the investigators at the survey site. If the investigators saw any unanswered questions, they asked the participants to complete them at the site. Questionnaires with missing values equal to or exceeding 15% and those failing to meet the inclusion criteria were excluded from the analysis. For questionnaires with missing values that were retained, the missing values were handled using multiple imputation methods.

### 2.5 Statistical analyses

Two investigators used EpiData 3.1 and Excel software to proofread and summarize the questionnaire data before using SPSS 26.0 to conduct statistical analysis. The frequency and constituent ratio (%) were calculated to use descriptive statistics to describe the overall situation of the sample. A univariate chi-square test was performed with the basic information of the sample as the independent variable and medication literacy as the dependent variable to analyze the relationship between the groups of demographic characteristics. The factors with statistical significance (*p* < 0.05) in the univariate analysis were included in the multiple linear regression analysis. Taking the medication literacy score as the dependent variable, the factors affecting the medication literacy of older adults were analyzed, with *p* < 0.05 considered statistically significant.

## 3 Results

### 3.1 Demographic characteristics

Of the 1,198 questionnaires distributed, 1,005 valid questionnaires were returned, with an effective recovery rate of 83.89%. Of the 10 administrative districts covered in Shenzhen, Longgang District accounted for the largest proportion of responses, representing 23.78%. It was followed by Baoan district at 22.89% and Nanshan District and Futian District at 14.83 and 10.45%, respectively. The proportion of males and females was relatively uniform, with males accounting for 48.16% and females for 51.84%. The average age of the sample was 67.26 ± 6.04 years. The education level of the participants was generally low, with 61.60% having a junior high school education or below. Most (86.37%) participants were married, and most (85.67%) did not live alone. Table 2 showed the participants’ demographic and clinical characteristics.

**Table 2:** Participant demographic and clinical characteristics (*N* = 1,005).

Variable	Group	*n*	%
Administrative district	Baoan	230	22.89
	Dapeng New Area	26	2.59
	Futian	105	10.45
	Guangming	48	4.78
	Longgang	239	23.78
	Longhua	90	8.96
	Luohu	71	7.06
	Nanshan	149	14.83
	Pingshan	23	2.29
	Yantian	24	2.39
Gender	Male	484	48.16
	Female	521	51.84
Age (years)	60–69	695	69.15
	70–79	255	25.37
	80–89	55	5.47
Education	Little or no literacy	82	8.16
	Primary school	211	21.00
	Junior high school	326	32.44
	High school/secondary school/technical high school	260	25.87
	Junior college	83	8.26
	Bachelor’s degree	40	3.98
	Master’s degree or above	3	0.30
Marital status	Unmarried	7	0.70
	Married	868	86.37
	Divorced	28	2.79
	Widowed	102	10.15
Living alone	Yes	144	14.33
	No	861	85.67
Monthly personal income (yuan)	≤1,000	243	24.18
	1,001–3,000	322	32.04
	3,001–5,000	303	30.15
	>5,000	137	13.63
Medical insurance	None	200	19.90
	Employee basic insurance	371	36.92
	Resident basic medical insurance	357	35.52
	Other	77	7.66
Number of comorbidities	0	379	37.71
	1	374	37.21
	2	190	18.91
	≥3	62	6.17
Disease duration (years)	0	395	39.30
	1–5	332	33.03
	6–10	164	16.32
	>10	114	11.34
Medications taken per day	0	393	39.10
	1	262	26.07
	2	211	21.00
	≥3	139	13.83
Frequency of hospitalizations in past year	0	719	71.54
	1	228	22.69
	2	48	4.78
	≥3	10	1.00
Adverse drug reaction history	Yes	116	11.54
	No	664	66.07
	Uncertain	225	22.39
The situation of contacting medical staff	Yes, regularly	173	17.21
	Yes, not regularly	328	32.64
	No	504	50.15

### 3.2 Grading of medication literacy score

Referring to the Chinese health literacy evaluation standards, a score of 80% or above on the questionnaire indicates that community residents possess medication literacy (13). Furthermore, referring to the WHO’s categorization of health literacy levels which was based on the research of Sørensen et al. (14), medication literacy could be stratified into three levels: ‘low’ (≤66%), ‘medium’ (>66–84%), and ‘high’ (>84%). The specific score ranges for each interval were shown in Table 3.

**Table 3:** Grading of medication literacy levels.

Level	Score proportion interval range	Score range
Low literacy	≤66%	20.00 ≦ S ≦ 66.00
Medium literacy	>66–84%	66.00 < S ≦ 84.00
High literacy	>84%	84.00 < S ≦ 100.00

### 3.3 Level of medication literacy in each administrative district

Taking administrative districts as subgroups, the medication literacy scores (including weight) of older adults were statistically analyzed and classified according to the evaluation criteria. The results showed that the mean medication literacy score of the participants in this study was at a medium level. Older adults in Nanshan District had the highest average score at 71.29 ± 14.87, while those in Yantian District had the lowest score at 67.91 ± 15.19.

Functional literacy was defined as possessing sufficient reading and writing skills to function effectively in the process of daily medication use. In the dimension of functional literacy, the scores of Dapeng New Area and Nanshan District were the highest at 1.47 ± 0.30 and 1.47 ± 0.33, respectively, while the lowest score was 1.41 ± 0.35 in Yantian district. The communication literacy dimension assessed whether the participants could seek information about drugs from different sources, actively interact with medical staff, and apply new information in the daily medication decision-making process. The scores for Nanshan District were the highest at 1.23 ± 0.32, while the scores of Yantian district were the lowest at 1.17 ± 0.32. In the dimension of critical literacy, which measured the ability to correctly and critically analyze drug information and exert control in the process of medication management. The scores of Nanshan District and Dapeng New District were the highest, at 0.87 ± 0.25 and 0.87 ± 0.23, respectively, while the lowest score was 0.82 ± 0.25 in Yantian district. These results indicated that critical thinking in the daily drug management of the older adults in Yantian district needed to be strengthened. Table 4 showed the dimension and total medication literacy scores for each district.

**Table 4:** Dimension and total medication literacy scores for each district.

District	Dimension score (mean ± SD)			Total score (100-mark system)	Level	Rank
	Functional literacy	Communication literacy	Critical literacy			
Baoan	1.44 ± 0.36	1.20 ± 0.34	0.85 ± 0.26	69.67 ± 16.44	Medium	7
Dapeng New	1.47 ± 0.30	1.22 ± 0.29	0.87 ± 0.23	71.25 ± 12.72	Medium	2
Futian	1.44 ± 0.36	1.20 ± 0.34	0.85 ± 0.26	69.81 ± 16.44	Medium	5
Guangming	1.43 ± 0.34	1.19 ± 0.32	0.84 ± 0.25	69.14 ± 14.62	Medium	9
Longgang	1.44 ± 0.37	1.20 ± 0.35	0.85 ± 0.26	69.65 ± 16.53	Medium	8
Longhua	1.44 ± 0.35	1.20 ± 0.33	0.85 ± 0.25	69.72 ± 15.44	Medium	6
Luohu	1.46 ± 0.33	1.22 ± 0.32	0.86 ± 0.25	70.98 ± 14.83	Medium	3
Nanshan	1.47 ± 0.33	1.23 ± 0.32	0.87 ± 0.25	71.29 ± 14.87	Medium	1
Pingshan	1.45 ± 0.33	1.21 ± 0.32	0.85 ± 0.24	70.41 ± 14.36	Medium	4
Yantian	1.41 ± 0.35	1.17 ± 0.32	0.82 ± 0.25	67.91 ± 15.19	Medium	10
Total	1.44 ± 0.37	1.20 ± 0.35	0.85 ± 0.26	69.71 ± 16.57	Medium	–

### 3.4 Correlation between each dimension score and medication literacy score

Spearman correlation analysis was used to analyze the correlation between the scores of the three dimensions of functional literacy, communication literacy, and critical literacy and the total medication literacy score. The results showed that the correlation coefficients between the scores of each dimension and the total score of the index system were significantly positively correlated at 0.875, 0.873, and 0.790 (*p* < 0.01). The scores of the three dimensions were also significantly positively correlated (*p* < 0.01), as shown in Table 5.

**Table 5:** Correlation analysis between total medication literacy score and each dimension score.

Dimension	Functional literacy	Communication literacy	Critical literacy	Medication literacy
Functional literacy	1	0.630*	0.542*	0.875*
Communication literacy	0.630*	1	0.563*	0.873*
Critical literacy	0.542*	0.563*	1	0.790*
Medication literacy	0.875*	0.873*	0.790*	1

*Significant at *p* < 0.01 (two-tailed).

### 3.5 Univariate analysis

In the univariate analysis, district, gender, age, education level, marital status, living status, personal monthly income, type of medical insurance, number of comorbidities, disease duration, number of daily medications, frequency of hospitalizations in the past year, history of adverse drug reactions (ADRs), and regular contact with medical staff were included as independent variables, and medication literacy was used as the dependent variable. The results showed that the factors significantly affecting medication literacy were district (*χ*^2^ = 66.839, *p* = 0.000), education level (*χ*^2^ = 184.423, *p* = 0.000), living alone (*χ*^2^ = 7.226, *p* = 0.027), personal monthly income (*χ*^2^ = 90.157, *p* = 0.000), type of medical insurance (*χ*^2^ = 82.719, *p* = 0.000), disease duration (*χ*^2^ = 18.174, *p* = 0.006), frequency of hospitalizations in the past year (*χ*^2^ = 19.196, *p* = 0.004), history of ADRs (*χ*^2^ = 30.614, *p* = 0.000), and regular contact with medical staff (*χ*^2^ = 37.197, *p* = 0.000). Table 6 showed the detailed results of the univariate analysis.

**Table 6:** Univariate analysis of medication literacy (*N* = 1,005).

Variable	Group	*n*	%	Medication literacy score (*x* ± *s*)	*χ* ^2^	*P*
Administrative district					66.839	0.000
	Baoan	230	22.89	69.67 ± 16.44		
	Dapeng New Area	26	2.59	71.25 ± 12.72		
	Futian	105	10.45	69.81 ± 16.44		
	Guangming	48	4.78	69.14 ± 14.62		
	Longgang	239	23.78	69.65 ± 16.53		
	Longhua	90	8.96	69.72 ± 15.44		
	Luohu	71	7.06	70.98 ± 14.83		
	Nanshan	149	14.83	71.29 ± 14.87		
	Pingshan	23	2.29	70.41 ± 14.36		
	Yantian	24	2.39	67.91 ± 15.19		
Gender					0.297	0.862
	Male	484	48.16	69.73 ± 16.33		
	Female	521	51.84	69.69 ± 16.80		
Age (years)					9.484	0.050
	60–69	695	69.15	69.45 ± 16.00		
	70–79	255	25.37	70.26 ± 17.88		
	80–89	55	5.47	70.46 ± 17.58		
Education					184.423	0.000
	Little or no literacy	82	8.16	53.36 ± 14.79		
	Primary school	211	21.00	63.08 ± 16.47		
	Junior high school	326	32.44	69.03 ± 15.39		
	High school/secondary school/technical high school	260	25.87	76.53 ± 13.26		
	Junior college	83	8.26	78.65 ± 14.63		
	Bachelor’s degree	40	3.98	80.56 ± 11.38		
	Master’s degree or above	3	0.30	73.90 ± 22.17		
Marital status					8.729	0.189
	Unmarried	7	0.70	62.85 ± 13.95		
	Married	868	86.37	70.06 ± 16.46		
	Divorced	28	2.79	61.13 ± 18.48		
	Widowed	102	10.15	69.55 ± 16.62		
Living alone					7.226	0.027
	Yes	144	14.33	66.27 ± 16.92		
	No	861	85.67	70.29 ± 16.45		
Monthly personal income (yuan)					90.157	0.000
	≤1,000	243	24.18	63.50 ± 17.93		
	1,001–3,000	322	32.04	67.15 ± 16.12		
	3,001–5,000	303	30.15	73.15 ± 14.79		
	>5,000	137	13.63	79.12 ± 12.47		
Type of medical insurance					82.719	0.000
	None	200	19.90	62.08 ± 17.21		
	Employee basic medical insurance	371	36.92	76.05 ± 14.30		
	Resident basic medical insurance	357	35.52	68.19 ± 16.33		
	Other	77	7.66	66.05 ± 15.25		
Number of comorbidities					9.083	0.169
	0	379	37.71	69.98 ± 17.18		
	1	374	37.21	70.79 ± 16.02		
	2	190	18.91	67.88 ± 16.32		
	≥3	62	6.17	67.20 ± 16.48		
Disease duration (years)					18.174	0.006
	0	395	39.30	70.24 ± 17.11		
	1–5	332	33.03	67.38 ± 16.48		
	6–10	164	16.32	70.83 ± 15.02		
	>10	114	11.34	73.05 ± 16.37		
Number of daily medications					7.388	0.286
	0	393	39.10	69.96 ± 17.21		
	1	262	26.07	68.44 ± 15.87		
	2	211	21.00	69.19 ± 16.78		
	≥3	139	13.83	72.19 ± 15.51		
Frequency of hospitalizations in the past year					19.196	0.004
	0	719	71.54	71.03 ± 16.69		
	1	228	22.69	66.63 ± 16.07		
	2	48	4.78	67.33 ± 15.24		
	≥3	10	1.00	56.13 ± 7.90		
ADR history					30.614	0.000
	Yes	116	11.54	70.23 ± 16.14		
	No	664	66.07	71.55 ± 16.49		
	Uncertain	225	22.39	64.02 ± 15.79		
The situation of contacting medical staff					37.197	0.000
	Yes, regularly	173	17.21	76.69 ± 15.48		
	Yes, not regularly	328	32.64	67.88 ± 15.26		
	No	504	50.15	68.50 ± 17.15		

### 3.6 Multiple linear regression analysis

To further explore the impact of influencing factors on medication literacy, multiple regression analysis was performed. The statistically significant variables in the univariate analysis (administrative district, education level, whether living alone, personal monthly income, type of medical insurance, disease duration, frequency of hospitalizations in the past year, ADR history, and regular contact with medical staff) were included as independent variables, and the total medication literacy score was used as the dependent variable. The method of independent variable assignment is shown in Table 7.

**Table 7:** Assignment of independent variables.

Independent variable	Description of assignment
Administrative district	1 = Baoan, 2 = Dapeng New Area, 3 = Futian, 4 = Guangming, 5 = Longgang, 6 = Longhua, 7 = Luohu, 8 = Nanshan, 9 = Pingshan, 10 = Yantian
Education	1 = Little or no literacy, 2 = Primary school, 3 = Junior high school, 4 = High school/secondary school/technical high school, 5 = Junior college, 6 = Bachelor’s degree, 7 = Master’s degree or above
Living alone	1 = Yes, 2 = No
Monthly personal income (yuan)	1 = ≤1,000, 2 = 1,001–3,000, 3 = 3,001–5,000, 4= > 5,000
Type of medical insurance	1 = No, 2 = Employee basic insurance, 3 = Resident basic medical insurance, 4 = Other
Disease duration (years)	1 = 0, 2 = 1–5, 3 = 6–10, 4= > 10
Frequency of hospitalizations in the past year	1 = 0, 2 = 1, 3 = 2, 4 = ≥3
ADR history	1 = Yes, 2 = No, 3 = Uncertain
The situation of contacting medical staff	1 = Yes, regularly, 2 = Yes, not regularly, 3 = No

**Figure 2:**
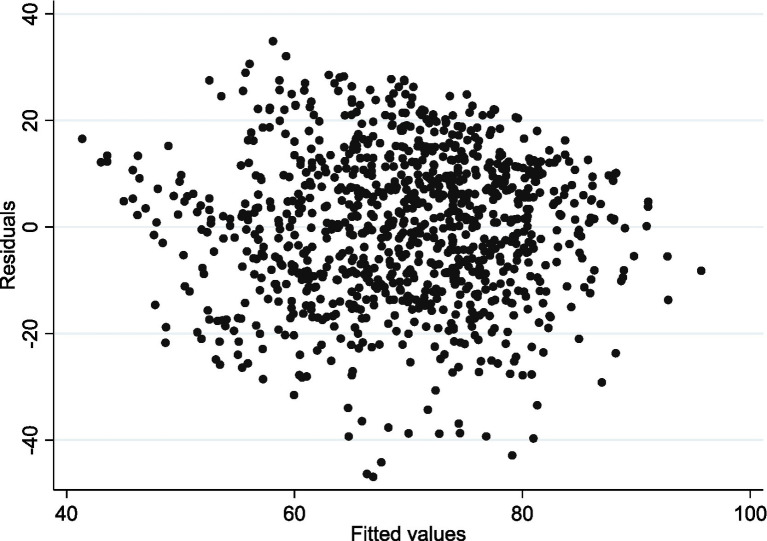
The residual plots.

**Figure 3:**
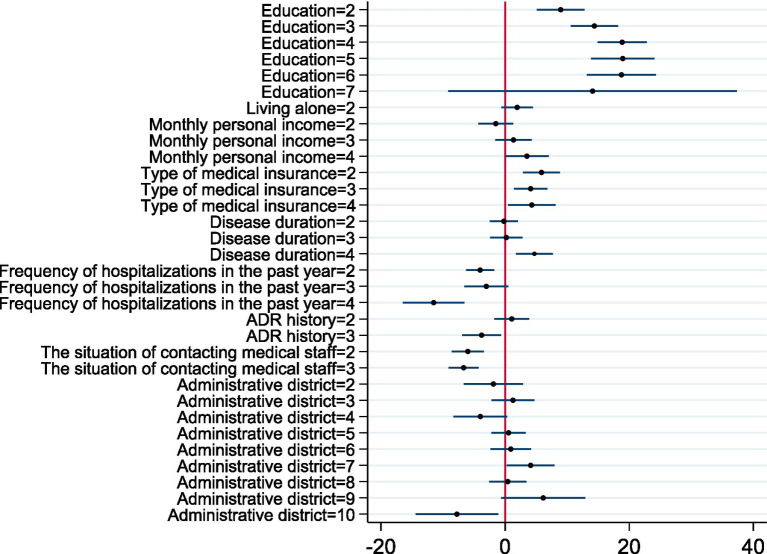
Visualization of regression coefficient forest plot.

Before conducting multiple linear regression, the tests for heteroskedasticity, multicollinearity, and normality were performed. The diagnostic results indicated that we could not reject the null hypothesis of no heteroscedasticity in the regression equation (*χ*^2^ = 405.82, *p* = 0.5211). Additionally, the Variance Inflation Factors (VIF) ranged from 1.05 to 3.82 (Table 8), suggesting the absence of severe multicollinearity. Therefore, neither heteroscedasticity nor multicollinearity significantly affected the regression results, and the use of multiple linear regression was appropriate. Meanwhile, the normality test results showed that the *p*-value for kurtosis was greater than the significance level of 0.05 (*χ*^2^ = 0.17, *p* = 0.6816), while the p-value for skewness was less than 0.05 (*χ*^2^ = 53.16, *p* = 0.0108), indicating that the data were not perfectly normally distributed. We also conducted linearity diagnostics, as shown in Figure 2. The points on the residual plot were randomly and uniformly distributed around the horizontal axis (where residuals were 0), with no discernible trend, indicating that the model as a whole conforms to the linear assumption. Based on these test results and for robustness considerations, we employed OLS regression with heteroscedasticity-robust standard errors for the multivariate regression analysis. Multiple linear regression showed that significant factors associated with medication literacy among older adults included educational level, type of medical insurance, disease duration, frequency of hospitalizations in the past year, ADR history, and the situation of contacting medical staff. All of these factors were statistically significant (*p* < 0.05). Detailed results were shown in Table 8. The residual plot was shown in Figure 2. Visualization of regression coefficient forest plot was shown in Figure 3.

**Table 8:** Multiple linear regression analysis of factors influencing medication literacy score (*N* = 1,005).

Variable	Group	Coefficient	Robust std. err.	*t*	*P*	95% CI	VIF
_cons		55.694	2.550	21.84	0.000	50.690 ~ 60.699	
Administrative district							
	Baoan						
	Dapeng New Area	−1.898	2.438	−0.78	0.436	−6.683 ~ 2.887	1.11
	Futian	1.257	1.766	0.71	0.477	−2.209 ~ 4.723	1.46
	Guangming	−3.997	2.213	−1.81	0.071	−8.341 ~ 0.347	1.23
	Longgang	0.545	1.412	0.39	0.700	−2.225 ~ 3.315	1.64
	Longhua	0.904	1.672	0.54	0.589	−2.378 ~ 4.186	1.33
	Luohu	4.105	1.968	2.09	0.037	0.241 ~ 7.967	1.29
	Nanshan	0.423	1.533	0.28	0.783	−2.586 ~ 3.432	1.57
	Pingshan	6.129	3.457	1.77	0.077	−0.6564 ~ 12.914	1.12
	Yantian	−7.772	3.397	−2.29	0.022	−14.439 ~ −1.105	1.12
Education							
	Little or no literacy						
	Primary school	8.940	1.960	4.56	0.000	5.093 ~ 12.787	2.92
	Junior high school	14.373	1.945	7.39	0.000	10.557 ~ 18.190	3.77
	High school/secondary school/technical high school	18.851	2.021	9.33	0.000	14.885 ~ 22.817	3.82
	Junior college	18.944	2.606	7.27	0.000	13.829 ~ 24.059	2.42
	Bachelor’s degree	18.730	2.840	6.59	0.000	13.156 ~ 24.304	1.80
	Master’s degree or above	14.081	11.856	1.19	0.235	−9.186 ~ 37.348	1.09
Living alone							
	Yes						
	No	1.930	1.302	1.48	0.139	−0.625 ~ 4.485	1.08
Monthly personal income (yuan)							
	≤1,000						
	1,001 ~ 3,000	−1.515	1.438	−1.05	0.292	−4.338 ~ 1.307	2.03
	3,001 ~ 5,000	1.337	1.496	0.89	0.372	−1.598 ~ 4.272	2.37
	>5,000	3.498	1.803	1.94	0.053	−0.040 ~ 7.035	2.19
Type of medical insurance							
	None						
	Employee basic medical insurance	5.853	1.518	3.86	0.000	2.875 ~ 8.831	2.75
	Resident basic medical insurance	4.105	1.377	2.98	0.003	1.404 ~ 6.807	2.05
	Other	4.289	1.962	2.19	0.029	0.439 ~ 8.139	1.37
Disease duration (years)							
	0						
	1 ~ 5	−0.215	1.155	−0.19	0.852	−2.482 ~ 2.052	1.49
	6 ~ 10	0.1763	1.340	0.13	0.895	−2.453 ~ 2.806	1.38
	>10	4.710	1.516	3.11	0.002	1.736 ~ 7.684	1.29
Frequency of hospitalizations in the past year							
	0						
	1	−4.031	1.156	−3.49	0.001	−6.299 ~ −1.764	1.23
	2	−3.0389	1.808	−1.68	0.093	−6.587 ~ 0.509	1.08
	≥3	−11.522	2.534	−4.55	0.000	−16.494 ~ −6.550	1.05
ADR history							
	Yes						
	No	1.0562	1.433	0.74	0.461	−1.757 ~ 3.869	2.41
	Uncertain	−3.796	1.597	−2.38	0.018	−6.930 ~ −0.661	2.43
The situation of contacting with medical staff							
	Yes, regularly						
	Yes, not regularly	−6.0218	1.321	−4.56	0.000	−8.615 ~ −3.429	2.09
	No	−6.696	1.235	−5.42	0.000	−9.120 ~ −4.272	2.13

*R*^2^ = 0.306, *F* = 17.820, *p* < 0.01.

## 4 Discussion and analysis

### 4.1 Overall level of medication literacy

A cross-sectional survey of the medication literacy of older adults in the 10 districts of Shenzhen showed that the overall average score was 69.71 ± 16.57, at a medium level. Low medication literacy was the most prevalent (*n* = 407, 40.50%), followed by medium (*n* = 366, 36.42%) and high literacy (*n* = 232, 23.08%). 40.50% of the respondents had a low level of medication literacy. Although the proportion of people with low medication literacy was lower than that reported by Mei et al. (15) in Central China, it still constituted the largest group in our study. Mei et al. (15) reported that 53.40% of the 412 participants had insufficient knowledge of rational drug use, showing it necessary to strengthen the medication knowledge of the older adults. This study investigated only the medication literacy level of older adults in Shenzhen. The survey results showed that drug knowledge levels in this group were lower than those of the general population (16). The results of this study indicated that compared with other age groups, the older adults had lower medication literacy (17), and more attention and research needed to improve their medication literacy. The assessment of “medium” medication literacy among the older adults in Shenzhen may appear contradictory given the city’s abundant educational resources and comprehensive health insurance system. In fact, this outcome results from the combined effects of Shenzhen’s unique demographic structure—as a city of immigrants with a large population of accompanying older adults from other provinces who generally have lower educational attainment (only 4.28% of the surveyed sample held a bachelor’s degree or higher)—the limited coverage of pharmaceutical science outreach services, and the inherent complexity of developing medication literacy.

### 4.2 Analysis of influencing factors

Education level was associated with medication literacy score. Compared to the illiterate sample group, the medication literacy scores of the sample groups with primary school, junior high school, senior high school, college, bachelor’s degree, and master’s degree or higher educational levels were, respectively, 8.940, 14.373, 18.851, 18.944, 18.730 and 14.081 points higher. The impact of education level on medication literacy has been demonstrated (18, 19), with studies reporting that individuals with a higher education level have a higher ability to access, understand, and calculate complex drug-related information and make correct medication decisions. By contrast, individuals with a low education level are often unable to obtain information about safe drug use from reliable sources and are more likely to be misled by pharmaceutical marketing and advertisements for drugs and healthcare products, leading to ADRs.

Medication literacy among older adults differed according to their type of medical insurance. Compared to the non-insurance sample group, the medication literacy scores of the sample groups with Employee basic medical insurance, Resident basic medical insurance, and other types of medical insurance were, respectively, 5.853, 4.105, and 4.289 points higher. The older adults with employee medical insurance had the highest level of medication literacy, followed by those with resident medical insurance, while those without medical insurance had the lowest level of medication literacy. The reason for these results may be that individuals with employee medical insurance have a higher economic status (20). As such, they do not ignore physical discomfort to reduce medical costs, and they pay attention to the safety and effectiveness of the medication process. Therefore, the medication literacy of the older adults without medical insurance should be better promoted.

Disease duration was identified as another factor relevant to the medication literacy of older adults. Compared to the group without disease duration, the medication literacy score of the sample group with a disease duration of more than 10 years was 4.710 points higher. Patients with over a decade of disease duration generally exhibit higher medication literacy, a phenomenon likely stemming from “adaptive learning” catalyzed by both the length of illness and the imperative of survival. Through internalizing knowledge, honing practical skills, psychologically adapting to their condition, and deepening their engagement with the healthcare system, they evolve from passive “patients” into active “managers” of their own health.

The frequency of hospitalizations in the past year was correlated with medication literacy levels, with participants who had been hospitalized three or more times in the past year having the lowest medication literacy score. Conversely, participants who had not been hospitalized over the past year exhibited the highest medication literacy score. Specifically, the medication literacy scores of the sample group with three or more hospitalizations in the past year were 11.522 points lower than those of the sample group without hospitalizations in the past year. The reason may be that the frequency of hospitalizations is correlated with the number of comorbidities. When patients with comorbidities and polypharmacy face complex medication plans and higher medication risks, they often find it difficult to fully understand drug information and to remember the correct use of each drug, and their medication literacy may be poor (21). In addition, patients with a high frequency of hospitalizations often feel helpless or lack confidence in their health status, and low self-efficacy affects patients’ attitudes and behaviors toward medication management. Therefore, reducing negative emotions related to their health conditions can improve their self-efficacy, thereby improving their medication literacy (22).

A history of ADRs was also associated with the medication literacy score of older adults. Compared to the sample group with a history of ADRs, the medication literacy score of the sample group uncertain about their own ADR history was 3.796 points lower. A plausible explanation for this phenomenon is that individuals with low medication literacy, when encountering health anomalies during the medication process, lack both the intrinsic cognitive ability to correctly associate these anomalies with the medication and the extrinsic action capability to actively seek verification to dispel doubts. This dual barrier of cognition and behavior traps them in the fog of ‘uncertainty’. A study have revealed a widespread lack of understanding among the general public regarding what ADRs are, and how to report them (23). This lack of knowledge is itself a manifestation of poor drug literacy, which directly leads to patients not knowing what to do next when they have doubts. A systematic review identified that individuals with low health literacy face difficulties in ‘problem recognition’ (including determining whether an ADR has occurred), necessitating specialized and simplified strategies to assist them (24).

The extent of contact with medical staff was another factor related to medication literacy levels in the older population. Compared to the sample group that maintained regular contact with medical staff, the medication literacy scores of the sample groups with irregular contact or no contact were, respectively, 6.022 and 6.696 points lower. Patients who can maintain regular contact with medical staff at home have higher medication literacy than others than those who do not maintain regular contact or do not maintain any contact. With the increase in frequency of communication between patients and medical staff, patients’ mastery of their health conditions and related drug knowledge is expected to gradually improve so that they gain a comprehensive understanding and achieve a higher level of medication literacy.

### 4.3 Countermeasures and suggestions

#### 4.3.1 Increase investment in medication literacy education for the public

The findings of this study strongly suggest that the government should adopt a series of measures to increase investment in public medication literacy education. First, relevant authorities should formulate clear policies and issue guidelines aimed at enhancing public medication literacy. This includes ensuring that healthcare professionals provide standardized and ongoing medication guidance to patients, thereby improving public trust in drug-related information. Furthermore, supported by dedicated funding and based on unified pharmaceutical knowledge curricula, medication literacy education should be incorporated into the national public health education system. To combat the spread of false drug information and improve the quality of public health services, the government must also strictly oversee access to drug information and safeguard the rights and interests of the older adults. Additionally, to prevent low-income and uninsured individuals from delaying medical care until their conditions become severe, the government should integrate them into the medical assistance system, providing access to low-cost or free essential medicines along with guidance on rational drug use.

Secondly, in light of the impact of low educational attainment among the older adults on their medication literacy, government authorities should mandate the differentiation of drug information leaflets into “professional” and “patient” versions. The patient-oriented version must be simplified to contain only essential information for safe use, including drug name, active ingredients, indications, contraindications, administration method, and dosage. To enhance readability and comprehension, authorities should require the use of plain language and, where appropriate, replace textual descriptions with standardized pictograms or schematic diagrams to visually convey critical instructions. Furthermore, the inclusion of a QR code on patient leaflets should be considered, allowing individuals to conveniently access supplementary audio or video explanations via mobile devices (13).

Finally, to enhance public awareness of rational medication use, multisectoral collaboration should be actively promoted. It is essential to mobilize all societal sectors to participate in medication literacy education, thereby expanding the social resources, channels, and platforms available for public engagement. Specific recommended measures include: (1) organizing large-scale campaigns to disseminate drug safety knowledge, and (2) encouraging districts and counties to regularly conduct localized medication literacy outreach and educational activities tailored to the needs of the older adults. Examples of such initiatives may involve pharmacists providing consultation services in community health stations, delivering education on preventing adverse drug reactions from polypharmacy, conducting comprehensive medication reviews related to insurance coverage, and implementing scheduled follow-up reminders through primary care channels.

#### 4.3.2 Establish and improve the medication literacy education service system

For older adults in the community, enhancing medication literacy constitutes a systematic endeavor that requires the establishment and reinforcement of a corresponding service support system.

First, a robust selection mechanism should be implemented to identify healthcare professionals for public medication literacy education. These individuals must undergo systematic and specialized training according to a standardized protocol to enhance their expertise, expand the scope, methods, and target groups of training, and deliver more comprehensive education—thus effectively improving patients’ medication literacy (25). In practice, healthcare providers should strengthen physician–patient communication and patient education, while conducting regular follow-ups to gain a sustained and thorough understanding of patients’ medication behaviors and promote rational drug use.

Second, a dedicated medication consultation hotline should be established. Traditional printed medication materials with fixed content often fail to meet the diverse information needs of different older populations. A medication consultation hotline would provide the older adults with access to professional pharmaceutical knowledge and a more convenient, efficient channel for obtaining drug-related information. Through this platform, pharmacists can deliver personalized medication guidance to older adults, thereby helping to prevent irrational medication use. Simultaneously, health departments can utilize the platform to promptly identify and control disease spread, reducing the overall medical burden.

Third, the adoption of “memory aid tools” should be actively promoted. With advancing age, memory decline among the older adults makes them prone to forgetting medications, especially when managing complex regimens. Healthcare departments may provide older adults patients with intelligent pillboxes equipped with voice reminder functions to prompt timely medication intake, thereby improving adherence.

#### 4.3.3 Improve individual medication management

Enhancing an individual’s medication literacy depends substantially on personal initiative. Individuals should cultivate scientifically grounded medication habits and maintain a clear understanding of the usage, dosage, timing, and contraindications of their prescribed drugs. To minimize missed or incorrect doses, technological aids—such as setting medication reminders and maintaining digital medication records on smartphones—should be actively utilized. Furthermore, individuals should learn to critically evaluate medication information, avoiding uncritical acceptance, and actively refine their cognitive approaches to medication use. Seeking professional assistance when needed is also essential to advancing one’s medication literacy.

Family trust and support play a vital role in improving medication management among older adult patients (26). Family members should increase their attentiveness to the needs and emotional well-being of older adults. By strengthening familial support, they can enhance medication supervision, facilitate effective information exchange, and provide sustained encouragement. This, in turn, can improve medication adherence, foster self-management capabilities, and contribute to the overall physical and mental health of older adult patients.

### 4.4 Limitations and prospects

It is essential to acknowledge some limitations of this study. The survey was confined to the older population in Shenzhen, and the representativeness of the sample may be constrained by the city’s specific socioeconomic structure. The exclusion of participants with “cognitive impairment” may lead to some selection bias. Self-report bias and unmeasured confounders may also have a certain impact on the results. Therefore, the caution is warranted when generalizing the findings to other regions or populations. Furthermore, the cross-sectional design precludes establishing causal relationships, making it difficult to determine the dynamic interaction between medication literacy levels and educational interventions. Future research could incorporate longitudinal tracking or intervention trials to further verify the actual effects of community pharmaceutical services on medication behaviors in older patients, and include cognitive function assessments to reduce potential bias. Additionally, the assessment framework could be iteratively refined to better accommodate the evaluation needs of diverse regions and populations.

## 5 Conclusion

A cross-sectional survey of medication literacy among older adults in community settings in Shenzhen indicated that the average medication literacy score fell within the medium range. Significant factors associated with medication literacy included educational level, type of medical insurance, disease duration, frequency of hospitalizations in the past year, history of ADRs, and whether regular contact with medical staff. Targeted educational interventions are recommended for subgroups with lower educational levels, lack of medical insurance, shorter disease duration, three or more hospitalizations in the past year, limited awareness of ADRs, and irregular contact with clinicians. Relevant authorities should develop tailored education plans aimed at these populations with lower medication literacy to effectively enhance their medication literacy level.

## Data Availability

The original contributions presented in the study are included in the article/supplementary material, further inquiries can be directed to the corresponding authors.
